# Correction: The foraging gene coordinates brain and heart networks to modulate socially cued interval timing in Drosophila

**DOI:** 10.1371/journal.pgen.1012140

**Published:** 2026-05-05

**Authors:** Hongyu Miao, Wenjing Li, Yongwen Huang, Woo Jae Kim

The second author’s name is spelled incorrectly. The correct name is: Wenjing Li. The correct citation is: Miao H, Li W, Huang Y, Kim WJ (2025) The foraging gene coordinates brain and heart networks to modulate socially cued interval timing in Drosophila. PLoS Genet 21(7): e1011752. https://doi.org/10.1371/journal.pgen.1011752
