# Supplementary figures and images for: The foraging gene coordinates brain and heart networks to modulate socially cued interval timing in Drosophila
Source: PLoS Genet. 2025 Jul 8;21(7):e1011752. doi: 10.1371/journal.pgen.1011752 (PMC12237022; doi:10.1371/journal.pgen.1011752)

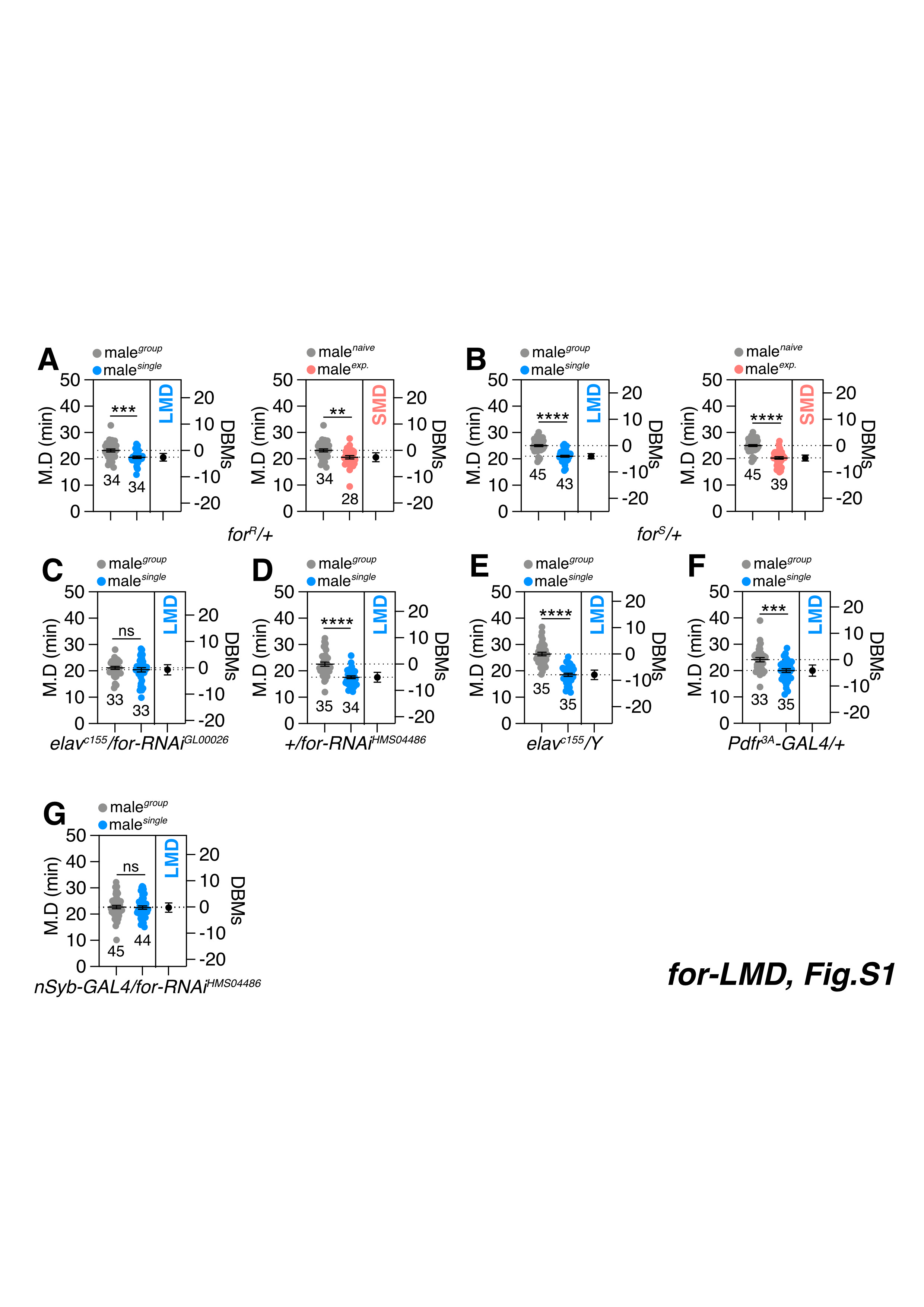

Supplement: S1 Fig — (A-B) LMD and SMD assays for forR/+ and forS/ + males. (C) LMD assay of flies expressing elavc155 drives together with for-RNAiGL00026. (D) LMD assay for for-RNAiHSM04486/ + . (E) LMD assay of flies for elavc155/Y. (F) LMD assay of flies for Pdfr2A-GAL4/ + . (G) LMD assay of flies expressing nSyb-GAL4 together with for-RNAi. (TIF) [file pgen.1011752.s001.tif]

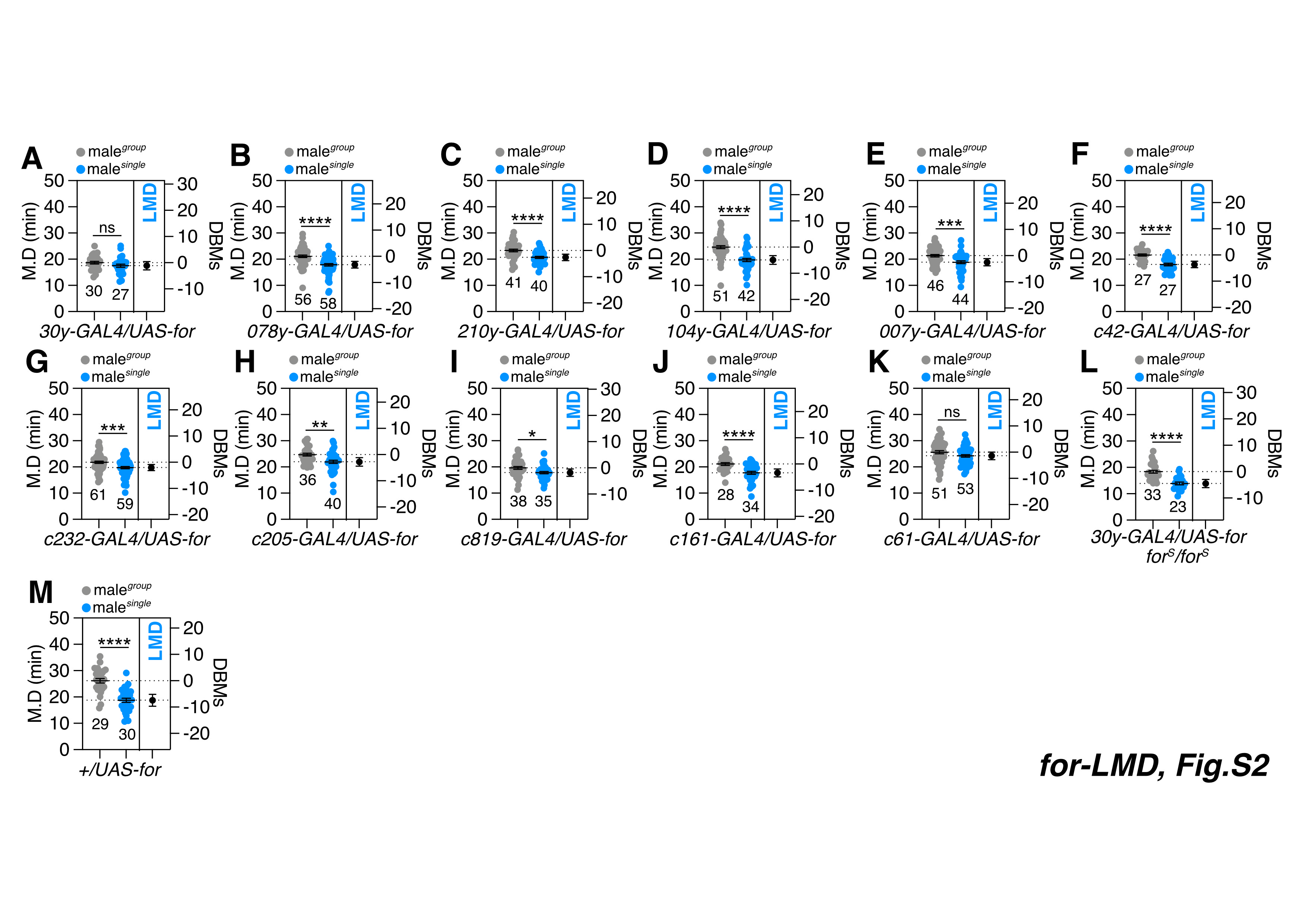

Supplement: S2 Fig — (A-K) LMD assays for male overexpressing foraging driven by subsets of neuronal cell drivers of the EB, 30y-GAL4(A), 078y-GAL4 (B), 210y-GAL4 (C), 104y-GAL4 (D), 007y-GAL4 (E), c42-GAL4 (F), c232-GAL4 (G), c205-GAL4 (H), c819-GAL4 (I), c161-GAL4 (J), and c61-GAL4 (K). (L) LMD assay for 30y-GAL4 drives for overexpression under fors homozygote. (M) LMD assay for UAS-for/ + . (TIF) [file pgen.1011752.s002.tif]

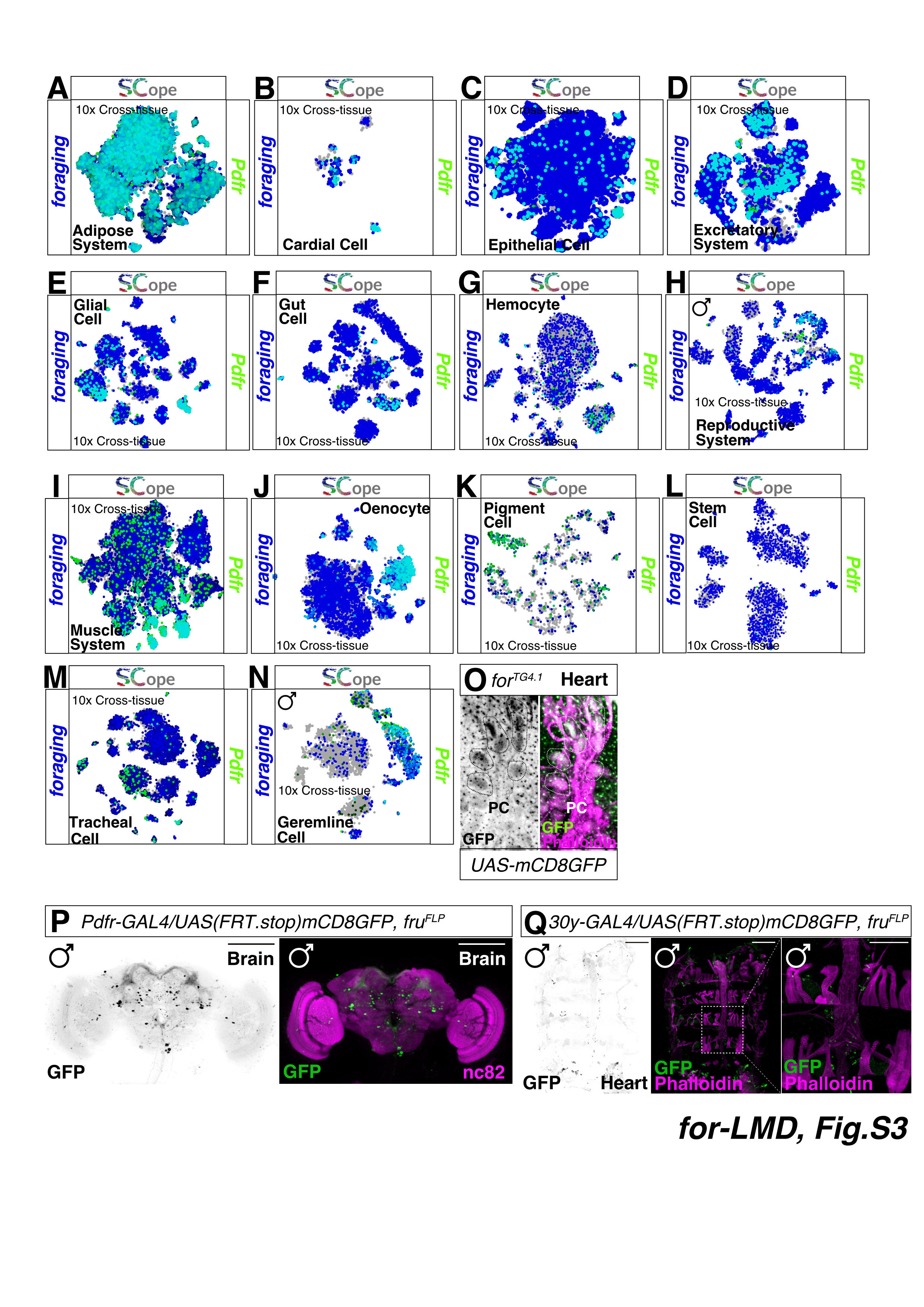

Supplement: S3 Fig — (A-N) SCOPE scRNA-seq datasets reveal cell clusters colored by expression of foraging (blue), Pdfr (green) in different tissues. (O) Male flies expressing the forTG4.1-GAL4 together with UAS-mCD8GFP were immunostained with anti-GFP (green) antibody and phalloidin (magenta). (P) Male flies brain expressing the UAS(FRT.stop)mCD8GFP; fruFLP together with Pdfr2A-GAL4 were immunostained with anti-GFP (green) and nc82 (magenta) antibodies. Scale bars represent 100 μm. (Q) Male flies heart expressing the UAS(FRT.stop)mCD8GFP; fruFLP together with 30y-GAL4 were immunostained with anti-GFP (green) antibody and phalloidin (magenta). Scale bars represent 100 μm. (TIF) [file pgen.1011752.s003.tif]

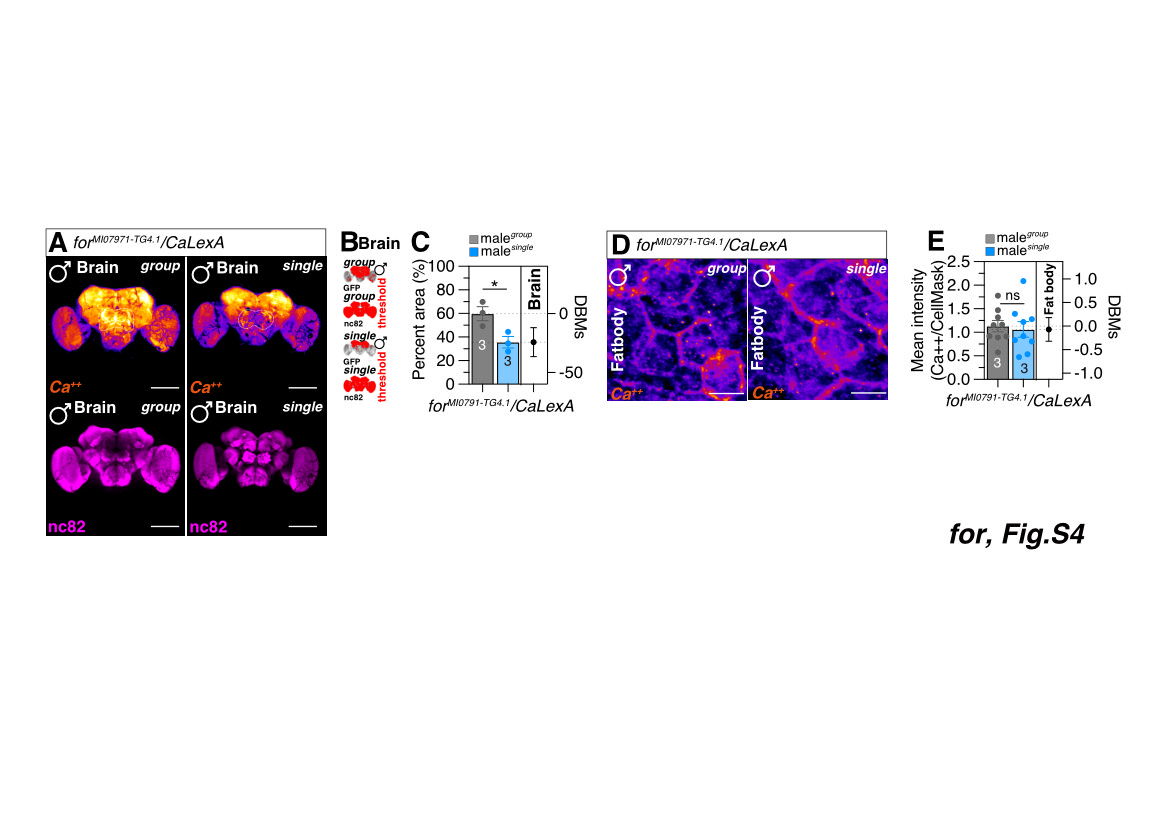

Supplement: S4 Fig — (A) Different levels of neural activity of the brain as revealed by the CaLexA system in group and single reared flies. Male flies expressing forMI01791-TG4.1 along with LexAop-CD8GFP(II); UAS-CaLexA, LexAop-CD2-GFP were dissected after 5 days of growth. The dissected brains were then immunostained with anti-GFP (green) and anti-nc82 (blue). GFP is pseudo-colored as “red hot”. Scale bars represent 100 μm in brain panels. (B) The GFP fluorescence (green) in male fly brain was processed using ImageJ software, where a threshold function was applied to distinguish fluorescence from the background. (C) Quantification of relative value for GFP fluorescence. (D) Different levels of intracellular calcium level of the fat body as revealed by the CaLexA system in group and single reared flies. Scale bars represent 25 μm in fat body panels. (E) Quantification of mean intensity for GFP fluorescence. (TIF) [file pgen.1011752.s004.tif]

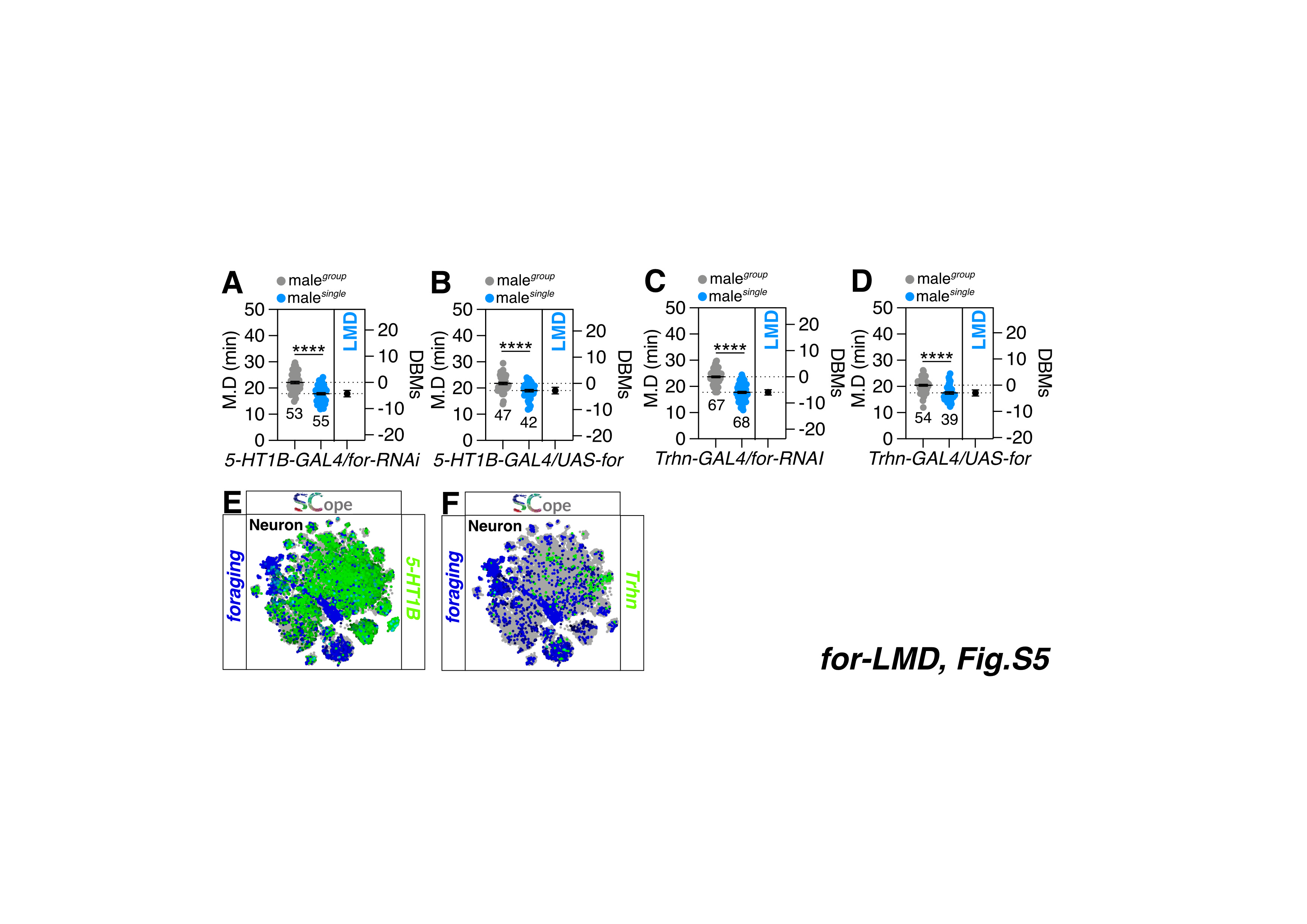

Supplement: S5 Fig — (A-B) LMD assays of flies expressing 5-HT1B-GAL4 drives together with for-RNAi (A) and UAS-for (B). (C-D) LMD assays of flies expressing Trhn-GAL4 drives together with for-RNAi (C) and UAS-for (D). (E) SCOPE scRNA-seq datasets reveal cell clusters colored by expression of foraging (blue), 5-HT1B (green) in neurons. (F) SCOPE scRNA-seq datasets reveal cell clusters colored by expression of foraging (blue), Trhn (green) in neurons. (TIF) [file pgen.1011752.s005.tif]

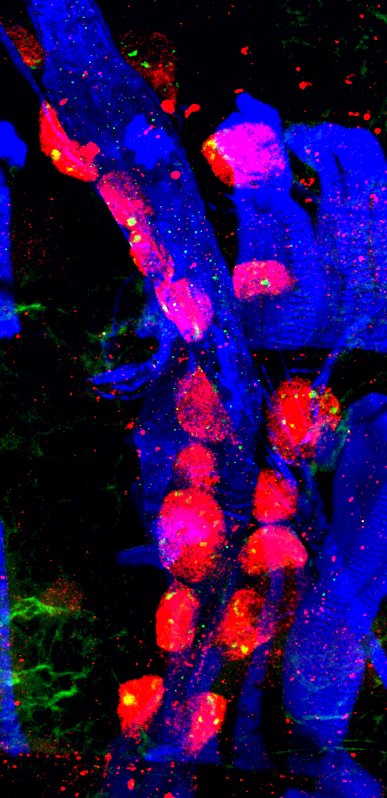

Supplement: S1 File — (JPG) [file pgen.1011752.s006.jpg]
