# Supplementary material for: The foraging gene coordinates brain and heart networks to modulate socially cued interval timing in Drosophila
Source: PLoS Genet. 2025 Jul 8;21(7):e1011752. doi: 10.1371/journal.pgen.1011752 (PMC12237022; doi:10.1371/journal.pgen.1011752)
Supplement: Attachment — Submitted filename: Review Plos genetics foraging gene mating 2025.docx [file pgen.1011752.s007.docx]

Manuscript number: RC-2024-02605

Corresponding author: Woo Jae, Kim

I was excited to read this paper as it addresses and brings together aspects of research around the role of the foraging gene that has remained refractory. The authors of the paper accomplish this with state-of-the-art techniques (e.g. split gal4, brain scRNAseq, outstanding design and control of Drosophila behavior-genetic experiments).  The phenotype under study, the interval timing of mating duration, is fascinating as it is an adaptive behaviour responsive to environmental input (e.g. previous social and mating experience, metabolic state, current social environment during the mating duration test). How our experience gets embedded into our biology from a gene regulatory and circuit perspective has broad significance to a variety of disciplines. Finally, because the foraging gene orthologues have been shown to play a role in behavioural tasks in eusocial insects, other invertebrates and vertebrates, the present study will be of interest to an audience that extends outside of Drosophila.

A role for the foraging gene in any aspect of D. melanogaster mating behavior has not been investigated.  Furthermore, little has been done to map the circuits that foraging is involved with for any of its pleiotropic traits, except for the latency of the larval nociception response (see Dason et al 2020, PNAS). Additionally, as mentioned by the authors, although foraging’s effects on GEI/plasticity in metabolic and behavioural responses of the rover/sitter variants have been shown, an understanding of how these effects might be regulated through tissue-specific functions is lacking. The authors of this manuscript are the first to identify foraging’s critical role in interval timing of mating duration, an important adaptive phenotype. Their results are convincing. They have identified two organs, subsets of cells in the heart and the ellipsoid body in the brain that together link foraging’s metabolic and neuronal/glial effects on mating duration. Furthermore, they have shown that foraging interacts with the “timing” gene pdfr in the brain and with the sex determination gene doublesex, in the heart to affect interval timing in mating. foraging’s role in plastic responses to the social environment in the heart is found when comparing male flies with and without social experience. Future research on this model might interrogate how environmental information to the heart gets transduced to the brain to affect interval timing on mating duration under different social and metabolic conditions. Future research could also address how foraging’s molecular complexity affects interval timing in mating behavior; foraging is a highly complex gene with 4 promoters, 21 transcripts and 8 protein isoforms. Mapping the modularity of the phenotypes under study, the effects of social environments, their tissue specificity and sex differences to foraging’s promotors, transcripts, proteins and gene regulatory networks, along with further circuit analysis will continue to develop the model studied in the current paper. I look forward to reading future papers from this group on this model.

Below are some further comments on your paper. Most of them are minor.

Introduction:

I like the introduction. It is thorough and well-written.

I want to point out that the title of the paper is specific, only representing the heart-specific function. Do the authors want the title to more broadly reflect the findings in the paper?

The paper Reaume CJ, Sokolowski MB, Mery F. 2011. A natural genetic polymorphism affects retroactive interference in Drosophila melanogaster. 2011 Proc Biol Sci. 278(1702):91-8. doi: 10.1098/rspb.2010.1337, may be of interest. It addresses retroactive interference/forgetting, linking, foraging behaviour, timing and memory in the rover/sitter variants.

Results:

Line 143, you state that “Rovers, which have one copy of the foraging gene (forR/-), exhibit higher levels of for-mRNA and PKG activity compared to sitters, who are homozygous for the loss-of-function allele (forS/forS)”. This is not correct. Rovers have 2 copies of the forR allelic variants-it is homozygous for forR. Furthermore, sitters are not loss of function alleles. They are hypomorphs and the sitter has two copies of the forS allele. Loss of function alleles of foraging are pupal lethal. See Allen et al 2017 Genetics. Please correct this. I don’t think these errors affect the results or interpretations in your paper.

Line 150-152, reword “neurons of sitter allele”. As you know alleles don’t have neurons. Say neurons carrying (or with) the sitter alleles or something like that.  Also, correct for when you say “neurons of rover allele” in the next sentence.

Line 154 “the axon terminal projections of sitter strains are altered”. Expand on this. Say how they are altered in their branching patterns at the larval neuromuscular junction (nmj). See also Dason et al 2019 J. Cell Science, and Dason and Sokolowski 2021 J. Neurogenet for further analyses that agree and expand on the 1999 Renger et al paper and provide information on foraging's pleiotropic effects at the synapse.

Line 161 Regarding the results of the forR/fors heterozygotes, I think that this is a very interesting result, a kind of co-dominance in the heterozygote. For a future study, it would be interesting to see if LMD and SMD behaviours are differentially regulated through each of foraging's 4 promoters.

Line 167-169, “Therefore, we hypothesize that an extremely high level of PKG activity specifically disrupts SMD, while an extremely low level of PKG activity specifically disrupts LMD behavior.” I think that this is an interesting idea. Are there other examples of this in the literature? I recollect that the fly dunce gene might work like this in learning and memory but can’t recall the paper.

Line 196, see also Kuntz S, Poeck B, Sokolowski MB, Strauss R. 2012 The visual orientation memory of Drosophila requires Foraging (PKG) upstream of Ignorant (RSK2) in ring neurons of the central complex. Learn Mem. 19(8):337-40. doi: 10.1101/lm.026369.

Lines 233 and 323, These are exciting results!

Line 393,  “with the rover variant specifically affecting SMD and the sitter variant affecting SMD.”  I think there is a typo one should say LMD.

Lines 443-445. There are many innovative aspects of this paper and lots of new knowledge that was not previously attainable because of the lack of tools available.

Line 513 reword, “The sample has been next be incubated” ?

Line 527 reword, the flies were reared in the group condition and dissected right after 5 days of rearing.

Line 529. the phrase “without after any action” is not necessary in this sentence or the one below this sentence.

Line 555-557, “Therefore, statistical comparisons were made between groups that were naïvely reared, sexually experienced and singly reared within each experiment.” The point “reared within each experiment” in this sentence makes perfect sense to me.

Figures: The SCope figures in the paper are difficult to read because there are no keys to tell us what the overlapping colours would be. Often in your figures, three genes are compared in for example, red, green and blue and when looking at the image, it is difficult to see which gene coexpresses with which other gene. This is true for most of your Scope representations. If you provided a key for colour of the overlaps (not just the colour for each gene on the axes), the results would be clear to the reader. The keys could be put in the figure captions or next to the figure. My experience is that when we make readers do too much work while looking at figures, many will give up. Another suggestion about the colours in your SCope figures is that they don’t work for people who are colour-blind. Whether this requires changes in your paper is, of course, up to the editor. Your images with gfp (green and purple) are fine in this regard.
