# Supplementary material for: The foraging gene coordinates brain and heart networks to modulate socially cued interval timing in Drosophila
Source: PLoS Genet. 2025 Jul 8;21(7):e1011752. doi: 10.1371/journal.pgen.1011752 (PMC12237022; doi:10.1371/journal.pgen.1011752)
Supplement: Attachment — Submitted filename: 2025-R2R-Foraging-PLOS-Genetics-WJK.docx [file pgen.1011752.s008.docx]

**Manuscript number:** RC-2024-02605

**Corresponding author:** Woo Jae, Kim

## 1. Point-by-point description of the revisions

**Reviewer #1**

***General Comments:*** *The authors have substantially revised the manuscript. The revised manuscript elucidates an important role of the Drosophila foraging (for) gene in bridging social cues and interval timing behaviors through dual neural and non-neural (cardiac) mechanisms. Combining behavioral assays, single-cell RNA sequencing, gene knockdown, and calcium imaging, the study provides novel insights into the genetic and environmental interplay underlying adaptive behaviors. While the manuscript has been significantly improved, I still have some minor concerns on the writing and interpretation of data.*

**Answer:** We sincerely thank Reviewer 1 for his/her insightful feedback and positive evaluation of our revised manuscript. We are particularly grateful for his/her acknowledgment of the novel insights into the *foraging* gene’s dual neural and non-neural mechanisms in regulating interval timing behaviors. During our revision, we serendipitously uncovered the critical role of heart-specific *foraging* expression in modulating social context-dependent calcium dynamics and LMD behavior, a discovery we have now emphasized and contextualized within the broader framework of adaptive behaviors. We have carefully addressed the reviewer’s suggestions to refine the writing clarity and strengthen data interpretation, ensuring that all conclusions align rigorously with the evidence presented. Thank you once again for your constructive critique and support of our work.

***Minor concerns:***

***Comment 1.*** *language issues. Remove claims such as 'novel'. Longer mating duration (LMD) has been repeatly dipicted such as in line 198, and wrongly stated in line 289.*

**Answer:** We identified two instances of the term "novel" in the INTRODUCTION and DISCUSSION sections, which we have rephrased as follows:

Original: "In this study, we present the novel finding that genes traditionally associated with foraging behavior are also involved in interval timing, a cognitive process crucial for decision-making and behavior."

Revised: "In this study, we demonstrate that genes traditionally associated with foraging behavior are also involved in interval timing, a cognitive process crucial for decision-making and behavior."

Original: "Our findings reveal a novel non-neuronal mechanism by which foraging modulates interval timing behaviors through its function in fru-positive heart cells."

Revised: "Our findings reveal a previously unrecognized non-neuronal mechanism by which foraging modulates interval timing behaviors through its function in fru-positive heart cells."

We removed ‘Longer mating duration (LMD)’ from line 289.

***Comment 2.*** *wrong lable of color in Figure 6E and 6G.*

**Answer:** We have corrected label error.

***Comment 3.*** *Regarding to the regular phenotype in forS/+ and forR/+ flies, but deficit LMD and SMD in forR/forS flies, I think an alternative explanation for this discrepency is that there are potential mutations other than the for gene in forR and forS flies, which are dominant in forR/forS flies, but recessive in forS/+ and forR/+ flies.*

**Answer:** We appreciate the reviewer's insightful suggestion and have incorporated the following addition to the Discussion section to address this point:

“While our findings support PKG-mediated mechanisms, we acknowledge the alternative possibility that background mutations in the *for^R^* or *for^s^* stocks might contribute to the observed transheterozygotes phenotype. Such mutations could behave recessively in heterozygotes but show dominant interactions in the *for^R^/for^S^* combination. Future studies using CRISPR-generated alleles could help resolve these competing explanations.”

**Reviewer #2**

***General Comments:*** *I thank the authors for the improvements in the manuscript. I am happy that within a short time they found Pdfr+ for+ (possibly fru+ too) cells, somewhat unexpectedly, in the adult heart, playing an important role in LMD. Given their complete absence from the earlier version (V1) of the manuscript, I urge the authors to ensure that these heart-related new findings are derived from meticulous and rigorous experiments with sufficient sample sizes and repeats, and conservative interpretations. There are still a few minor errors (like Pdfr3A-gal4 in Fig 3 should be Pdfr-2A-Gal4). Lastly, I fail to understand in Fig 6. where does the RFP signal come from - it's unclear from the written genotypes. Please clarify if you measured integrated signal density or just the area of the manually chosen ROI to quantify CaLexA - it's not clear from the "Mat & Met".*

**Answer:** We sincerely thank the reviewer for their thoughtful evaluation and constructive feedback, which have significantly strengthened our manuscript. Below, we address their remaining concerns:

**1. Rigor of Heart-Related Findings**

We appreciate the reviewer’s emphasis on meticulous experimental design. The heart-specific *Pdfr+/for+* findings are supported by:

- **Robust sample sizes**: Behavioral assays (n > 20 males per genotype with different RNAi strains), calcium imaging (n = 5 hearts per condition), and scRNA-seq validation (multiple tissue-specific clusters).

- **Independent replicates**: All key experiments (e.g., Hand-GAL4 knockdown, CaLexA imaging) were repeated across at least two biological replicates.

- **Conservative interpretation**: We note the unexpected nature of these findings and have carefully framed our conclusions to emphasize the necessity of future mechanistic investigations (Discussion, paragraph 4). Given that knockdown of *foraging* in other tissues had no effect on LMD, our results strongly suggest a heart-specific role for *foraging* in regulating rival-induced prolonged mating duration.

**2. Correction of Pdfr-GAL4 Labeling**

We sincerely apologize for the labeling inconsistency in the original manuscript. The designation "*Pdfr^3A^-GAL4*" in Figure 3 and S3 has been corrected to "*Pdfr-GAL4*" throughout the text and figures. This GAL4 line, originally generated in the Paul Taghert lab, was historically termed “*Pdfr(3A)-GAL4*" (X-chromosome insertion) and deposited at the Bloomington Drosophila Stock Center (BDSC #33070). While this line is no longer available through BDSC, its use and nomenclature as "*Pdfr-GAL4*" are well-documented in prior studies (Górska-Andrzejak et al. 2018; Hidalgo et al. 2023). We have adopted this standardized naming convention to align with established literature and ensure clarity.

**3. Clarification of RFP Signal in Figure 6**

The RFP signal in Figure 6 originates from the UAS-mCD8RFP transgene from X chromosome (BDSC #32229), which labels cell membranes independently of CaLexA-driven GFP. This signal served as a counterstain to identify pericardial cell boundaries. We have clarified this in the revised figure legend and Methods section as below:

“CalexA assay for *Hand-GAL4* together with *UAS-mCD8RFP, lexAop-mCD8GFP;* l*exAop-mCD8GFP; UAS-CaLexA, lexAop-CD2-GFP* of genetic control (*empty-RNAi*, up) and group male flies with knocking down *foraging* (*for-RNAi*, bottom). RFP (*UAS-mCD8RFP*) labels cell membranes independently of CaLexA-GFP.” (**Figure legends**)

“To independently label cell membranes from CaLexA-driven GFP signals, we generated a transgenic strain (*UAS-mCD8RFP, lexAop-mCD8GFP* (X); *LexAop-CD8GFP* (II); *UAS-CaLexA, LexAop-CD2GFP* (III) by integrating BDSC stock #32229 and #66542. This design leverages distinct expression systems (UAS vs. LexAop) to separate RFP (membrane marker) and GFP (calcium activity reporter) signals.” **(Methods)**

Figure 6A/D/F is annotated with ‘*Hand-GAL4/CaLexA, UAS-mCD8RFP’* to clarify the experimental design and genetic tools employed.

**4. CaLexA Quantification Methodology**

CaLexA signals were quantified using **integrated signal density** (summed pixel intensity within manually defined ROIs), not merely ROI area as per Kayser et al. (Kayser et al. 2014). This method is now explicitly described in the Methods under “**Quantitative analysis of fluorescence intensity**” and cited in the Figure 6 legend. Following reviewer’s suggestion, we have added sentence explaining details of our measure as below:

“CaLexA signals were quantified using ‘integrated signal density’ (summed pixel intensity within manually defined ROIs), not merely ROI area.”

We are grateful for the reviewer’s diligence in ensuring the clarity and rigor of our work. Their insights have been invaluable in refining this study, and we hope these revisions fully address their concerns.

**Reviewer #3**

***General Comments:*** *I was excited to read this paper as it addresses and brings together aspects of research around the role of the foraging gene that has remained refractory. The authors of the paper accomplish this with state-of-the-art techniques (e.g. split gal4, brain scRNAseq, outstanding design and control of Drosophila behavior-genetic experiments). The phenotype under study, the interval timing of mating duration, is fascinating as it is an adaptive behaviour responsive to environmental input (e.g. previous social and mating experience, metabolic state, current social environment during the mating duration test). How our experience gets embedded into our biology from a gene regulatory and circuit perspective has broad significance to a variety of disciplines. Finally, because the foraging gene orthologues have been shown to play a role in behavioural tasks in eusocial insects, other invertebrates and vertebrates, the present study will be of interest to an audience that extends outside of Drosophila.*

*A role for the foraging gene in any aspect of D. melanogaster mating behavior has not been investigated. Furthermore, little has been done to map the circuits that foraging is involved with for any of its pleiotropic traits, except for the latency of the larval nociception response (see Dason et al 2020, PNAS). Additionally, as mentioned by the authors, although foraging’s effects on GEI/plasticity in metabolic and behavioural responses of the rover/sitter variants have been shown, an understanding of how these effects might be regulated through tissue-specific functions is lacking. The authors of this manuscript are the first to identify foraging’s critical role in interval timing of mating duration, an important adaptive phenotype. Their results are convincing. They have identified two organs, subsets of cells in the heart and the ellipsoid body in the brain that together link foraging’s metabolic and neuronal/glial effects on mating duration. Furthermore, they have shown that foraging interacts with the “timing” gene pdfr in the brain and with the sex determination gene doublesex, in the heart to affect interval timing in mating. foraging’s role in plastic responses to the social environment in the heart is found when comparing male flies with and without social experience. Future research on this model might interrogate how environmental information to the heart gets transduced to the brain to affect interval timing on mating duration under different social and metabolic conditions. Future research could also address how foraging’s molecular complexity affects interval timing in mating behavior; foraging is a highly complex gene with 4 promoters, 21 transcripts and 8 protein isoforms. Mapping the modularity of the phenotypes under study, the effects of social environments, their tissue specificity and sex differences to foraging’s promotors, transcripts, proteins and gene regulatory networks, along with further circuit analysis will continue to develop the model studied in the current paper. I look forward to reading future papers from this group on this model.*

Below are some further comments on your paper. Most of them are minor.

**Response to Reviewer’s General Comments**: We sincerely thank the reviewer for their enthusiastic and insightful evaluation of our work, as well as their recognition of its significance and innovation. We are deeply gratified by their appreciation for the novel contributions of our study, particularly in bridging gaps in understanding the *foraging* gene’s role in adaptive behaviors and its pleiotropic mechanisms. Below, we highlight key points of alignment with the reviewer’s thoughtful analysis:

1. **Novelty and Technical Rigor**: We are grateful for the acknowledgment of our use of state-of-the-art approaches (e.g., split-Gal4, single-cell RNA sequencing) to map *foraging*’s roles in interval timing. The reviewer rightly emphasizes that this is the first study to link *foraging* to mating duration in *Drosophila* and to delineate its tissue-specific functions in both neural (ellipsoid body) and non-neural (heart) contexts—a significant advance beyond prior work focused on larval nociception.
2. **Interdisciplinary Relevance**: We agree that the adaptive nature of interval timing—integrating social experience, metabolic state, and environmental cues—holds broad implications for understanding how genes encode plasticity across species. The reviewer’s emphasis on the conserved role of *foraging* orthologues in eusocial insects and vertebrates underscores the translational potential of our findings.
3. **Mechanistic Insights**: The identification of *foraging*’s interaction with *pdfr* (in brain circuits) and *doublesex* (in heart cells) opens new avenues to dissect how genetic and environmental inputs converge on interval timing. We appreciate the reviewer’s recognition of this dual mechanism as a critical step toward unraveling gene-environment interplay in adaptive behaviors.
4. **Future Directions**: We enthusiastically endorse the reviewer’s proposed future directions, including elucidating heart-to-brain communication pathways and dissecting *foraging*’s molecular complexity (e.g., isoform-specific roles, promoter usage). These align with our ongoing efforts to explore how environmental signals are transduced across tissues and how *foraging*’s modular architecture shapes behavioral plasticity.

Once again, we thank the reviewer for their generous appraisal of our work and their constructive vision for its future extensions. Their insights have profoundly shaped our perspective, and we look forward to building on this foundation in subsequent studies.

***Comment 1:*** *Introduction: I like the introduction. It is thorough and well-written. I want to point out that the title of the paper is specific, only representing the heart-specific function. Do the authors want the title to more broadly reflect the findings in the paper? The paper Reaume CJ, Sokolowski MB, Mery F. 2011. A natural genetic polymorphism affects retroactive interference in Drosophila melanogaster. 2011 Proc Biol Sci. 278(1702):91-8. doi: 10.1098/rspb.2010.1337, may be of interest. It addresses retroactive interference/forgetting, linking, foraging behaviour, timing and memory in the rover/sitter variants.*

**Answer:** We sincerely thank the reviewer for their constructive suggestion to broaden the title’s scope. In response, we have revised the title to:

**“The *foraging* Gene Coordinates Brain and Heart Networks to Modulate Socially Cued Interval Timing in *Drosophila*.”**

This revision better encapsulates the dual neural and cardiac mechanisms uncovered in our study, emphasizing how foraging integrates social cues across tissues to regulate adaptive timing behaviors. We believe this title now comprehensively reflects the manuscript’s central findings while maintaining clarity and precision. We have incorporated a citation to Reaume et al.'s excellent study (Reaume et al. 2011) in our manuscript, where it provides valuable context for rover and sitter allelic differences. Their work offers important insights into genetic polymorphism of *foraging*’s natural allele which strengthens the interpretation of our findings regarding their distinct roles on LMD and/or SMD behaviors. Thank you for guiding us toward improving the manuscript’s accessibility and impact.

***Comment 2:*** *Results: Line 143, you state that “Rovers, which have one copy of the foraging gene (forR/-), exhibit higher levels of for-mRNA and PKG activity compared to sitters, who are homozygous for the loss-of-function allele (forS/forS)”. This is not correct. Rovers have 2 copies of the forR allelic variants-it is homozygous for forR. Furthermore, sitters are not loss of function alleles. They are hypomorphs and the sitter has two copies of the forS allele. Loss of function alleles of foraging are pupal lethal. See Allen et al 2017 Genetics. Please correct this. I don’t think these errors affect the results or interpretations in your paper.*

**Answer:** We sincerely thank the reviewer for identifying this critical error in our genetic descriptions and for directing us to the foundational work by Allen et al. (2017) (Allen et al. 2017). We have carefully revised the manuscript to address this oversight as follows:

Correction of Genetic Terminology: The original statement in Line 143 has been revised to:

“Natural rover (*forᴿ/forᴿ*) and sitter (*forˢ/forˢ*) strains exhibit allelic differences in *foraging* gene expression, with rovers showing elevated PKG activity compared to sitters due to the hypomorphic nature of the *forˢ* allele (Allen et al., 2017).”

We removed the incorrect reference to “loss-of-function” for the *forˢ* allele, replacing it with “hypomorphic” to accurately reflect its partial functional reduction. We confirm that this error did not affect the experimental results or interpretations, as the behavioral assays and molecular analyses were conducted using the correct genotypes (homozygous forᴿ/forᴿ and forˢ/forˢ strains). Thank you again for your meticulous review, which has strengthened the accuracy and rigor of our manuscript.

***Comment 3:*** *Line 150-152, reword “neurons of sitter allele”. As you know alleles don’t have neurons. Say neurons carrying (or with) the sitter alleles or something like that. Also, correct for when you say “neurons of rover allele” in the next sentence.*

**Answer:** We sincerely thank the reviewer for highlighting this important inaccuracy in our phrasing. We have revised the text to clarify that neurons are derived from flies carrying specific *foraging* alleles, not that alleles themselves "have" neurons. The corrections are as follows:

“Neurons from sitter-allele homozygotes (*forˢ/forˢ*) display higher levels of spontaneous activity and exhibit exaggerated responses to stimulation, known as excessive evoked firing, which are not observed in neurons from rover-allele homozygotes (*forᴿ/forᴿ)*.”

Thank you again for your meticulous attention to detail, which has improved the clarity and rigor of our work.

***Comment 4:*** *Line 154 “the axon terminal projections of sitter strains are altered”. Expand on this. Say how they are altered in their branching patterns at the larval neuromuscular junction (nmj). See also Dason et al 2019 J. Cell Science, and Dason and Sokolowski 2021 J. Neurogenet for further analyses that agree and expand on the 1999 Renger et al paper and provide information on foraging's pleiotropic effects at the synapse.*

**Answer:** The *foraging* gene (*for*), encoding a cGMP-dependent protein kinase (PKG), exerts pleiotropic effects on synaptic structure and function at the larval NMJ in *Drosophila*. Sitter strains (*forˢ*), characterized by reduced PKG activity, exhibit distinct alterations in axon terminal branching patterns compared to rover strains (*forᴿ*). These differences are rooted in synaptic morphology, vesicle dynamics, and cytoskeletal regulation, as supported by studies from Renger et al. (1999), Dason et al. (2019), and Dason & Sokolowski (2021) (Renger et al. 1999; Dason et al. 2020; Dason and Sokolowski 2021). Below, we synthesize these findings to clarify how *forˢ* alleles alter NMJ architecture:

“The foraging gene’s pleiotropic roles in synaptic structure, vesicle dynamics, and neuronal-glial interactions underscore its importance in balancing energy allocation and synaptic plasticity. These findings provide a framework for understanding how genetic variation in for (PKG) shapes adaptive behaviors through NMJ remodeling [43,44].”

***Comment 5:*** *Line 161 Regarding the results of the forR/fors heterozygotes, I think that this is a very interesting result, a kind of co-dominance in the heterozygote. For a future study, it would be interesting to see if LMD and SMD behaviours are differentially regulated through each of foraging's 4 promoters.*

**Answer:** We thank the reviewer for highlighting the intriguing co-dominant effects in *forᴿ/forˢ* heterozygotes. In future work, we will:

1. Generate promoter-specific CRISPR mutants of *foraging* (P1–P4) to dissect their roles in LMD and SMD.
2. Test whether distinct promoters drive isoform expression in neural (*Pdfr*+) versus cardiac (*fru*+) circuits.
3. Map promoter activity under varying social/metabolic conditions to link environmental inputs to isoform regulation.

This will clarify how *foraging*’s promoter architecture balances plasticity (LMD) and homeostasis (SMD), addressing evolutionary trade-offs in pleiotropic genes.

***Comment 6:*** *Line 167-169, “Therefore, we hypothesize that an extremely high level of PKG activity specifically disrupts SMD, while an extremely low level of PKG activity specifically disrupts LMD behavior.” I think that this is an interesting idea. Are there other examples of this in the literature? I recollect that the fly dunce gene might work like this in learning and memory but can’t recall the paper.*

**Answer:** The reviewer raises an intriguing question about whether other genes exhibit dose-dependent behavioral effects similar to the *foraging* gene’s PKG activity thresholds (high PKG disrupting SMD, low PKG disrupting LMD). Below are examples from the literature that align with this phenomenon, including the *dunce* gene in *Drosophila* system:

The *dunce (dnc)* gene in *Drosophila melanogaster* provides a relevant example of how differential enzyme activity levels or isoform-specific functions can influence distinct memory processes. Classical *dnc* mutants (e.g., *dnc¹*) exhibit **reduced short-term memory (STM)** due to globally elevated cAMP levels caused by impaired phosphodiesterase (PDE) activity. However, isoform-specific mutants (e.g., *dnc^Δ143^*, affecting the Dnc^PA^ isoform) paradoxically show **enhanced STM**, demonstrating that reduced activity of specific isoforms can improve memory performance. Conversely, other isoforms (e.g., Dnc^PB^) also regulate memory in opposing ways, highlighting that spatial expression (e.g., somatic vs. synaptic compartments) and isoform-specific activity critically determine behavioral outcomes. This aligns with the hypothesis that extreme levels of enzyme activity (high or low) can disrupt distinct memory forms, as seen with PKG in the reviewer’s comment. The study underscores the importance of **compartmentalized cAMP signaling** and isoform diversity in balancing memory processes, providing a precedent for activity-level-dependent behavioral effects. **Reference:** Hasselmann, T. (2023). *Not a dunce after all?-Dunce isoforms show differential localisation and influence memory in Drosophila melanogaster larvae* (Doctoral dissertation, Universität zu Köln).

***Comment 7:*** *Line 196, see also Kuntz S, Poeck B, Sokolowski MB, Strauss R. 2012 The visual orientation memory of Drosophila requires Foraging (PKG) upstream of Ignorant (RSK2) in ring neurons of the central complex. Learn Mem. 19(8):337-40. doi: 10.1101/lm.026369.*

**Answer:** We have integrated the work of Kuntz et al., which investigates the role of foraging behavior in memory processes, to further support and contextualize this hypothesis.

***Comment 8:*** *Lines 233 and 323, These are exciting results!*

**Answer:** Thank you!!

***Comment 9:*** *Line 393, “with the rover variant specifically affecting SMD and the sitter variant affecting SMD.” I think there is a typo one should say LMD.*

**Answer:** We have corrected this error.

***Comment 10:*** *Lines 443-445. There are many innovative aspects of this paper and lots of new knowledge that was not previously attainable because of the lack of tools available.*

**Answer:** Thank you sincerely for your kind and encouraging feedback. We deeply appreciate your recognition of the innovative aspects of our work and your acknowledgment of the new knowledge made possible by recent advancements in tools and methodologies. It is immensely gratifying to know that our contributions resonate with your perspective, and we are thrilled that the novel approaches outlined in the paper could address gaps previously constrained by technological limitations.

Your thoughtful engagement with our work motivates us to continue pushing boundaries in this field. Thank you once again for your support and for highlighting the significance of our findings.

***Comment 11:*** *Line 513 reword, “The sample has been next be incubated” ?*

**Answer:** Thank you for your valuable feedback. We have carefully rephrased the sentence in the Methods section to enhance clarity and precision. The revised text now reads:

"The samples were first incubated overnight at 4°C with primary antibodies diluted in 1% PBT. Subsequently, fluorophore-conjugated secondary antibodies were applied and incubated for one hour at room temperature."

This adjustment better emphasizes the sequential steps of the protocol while maintaining technical accuracy. We appreciate your attention to detail and your constructive suggestions, which have strengthened the manuscript. Thank you again for your thoughtful review.

***Comment 12:*** *Line 527 reword, the flies were reared in the group condition and dissected right after 5 days of rearing.*

**Answer:** We have rephrased this part.

***Comment 13:*** *Line 529. the phrase “without after any action” is not necessary in this sentence or the one below this sentence.*

**Answer:** We have removed those words.

***Comment 14:*** *Line 555-557, “Therefore, statistical comparisons were made between groups that were naïvely reared, sexually experienced and singly reared within each experiment.” The point “reared within each experiment” in this sentence makes perfect sense to me.*

**Answer:** Thank you for your positive feedback on the clarity of the phrasing regarding our statistical comparisons (Lines 555–557). We are grateful for your acknowledgment of our efforts to ensure methodological rigor. To strengthen our analysis, we employed robust statistical frameworks, including estimation statistics, to present results with greater transparency and precision.

Our approach builds on the methodology established in our prior work on SMD behavior published in *PLOS Genetics* (Lee et al. 2023), where we first refined these analytical techniques. For consistency and comparability, we adhered to the same rigorous standards in this study.

We sincerely appreciate your attentive review and your recognition of the logical coherence in our experimental design.

***Comment 15:*** *Figures: The SCope figures in the paper are difficult to read because there are no keys to tell us what the overlapping colours would be. Often in your figures, three genes are compared in for example, red, green and blue and when looking at the image, it is difficult to see which gene coexpresses with which other gene. This is true for most of your Scope representations. If you provided a key for colour of the overlaps (not just the colour for each gene on the axes), the results would be clear to the reader. The keys could be put in the figure captions or next to the figure. My experience is that when we make readers do too much work while looking at figures, many will give up. Another suggestion about the colours in your SCope figures is that they don’t work for people who are colour-blind. Whether this requires changes in your paper is, of course, up to the editor. Your images with gfp (green and purple) are fine in this regard.*

**Answer:** Thank you for your thoughtful suggestions regarding the clarity and accessibility of our SCope figures. We sincerely appreciate your attention to detail and your advocacy for improving readability, especially for readers with color vision deficiencies.

1. **Color Adjustments for Accessibility**:
   As part of our initial revisions, we updated the color schemes in select SCope figures (e.g., two-color combinations) to blue and green to better accommodate color-blind readers. However, we retained the original formats for figures such as **Fig. 3B/D/F/I/J**, **Fig. S3A-N**, and **Fig. 5A** to maintain consistency with established visual conventions in prior analyses.
2. **Addition of Color Overlap Keys**:
   In direct response to your feedback, we have now included **detailed color-code keys** in the captions of all relevant SCope figures with three colors. These keys explicitly define:
   - The gene(s) represented by each axis color (e.g., red, green, blue).
   - The meaning of overlapping color combinations (e.g., yellow for red/green overlap, cyan for green/blue overlap).
   - A brief explanation of coexpression patterns.

We agree that these additions will significantly reduce reader effort and enhance interpretability of the data.

3. **Openness to Further Adjustments**:
   If the editor recommends additional modifications (e.g., further color adjustments for accessibility), we are fully prepared to implement them.

Thank you again for your constructive critique, which has undoubtedly strengthened the clarity and inclusivity of our work.

**REFERENCES**

Allen AM, Anreiter I, Neville MC, Sokolowski MB. 2017. Feeding-Related Traits Are Affected by Dosage of the foraging Gene in Drosophila melanogaster. Genetics. 205(2):761–773. doi:10.1534/genetics.116.197939.

Dason JS, Cheung A, Anreiter I, Montemurri VA, Allen AM, Sokolowski MB. 2020. Drosophila melanogaster foraging regulates a nociceptive-like escape behavior through a developmentally plastic sensory circuit. Proc Natl Acad Sci. 117(38):23286–23291. doi:10.1073/pnas.1820840116.

Dason JS, Sokolowski MB. 2021. A cGMP-dependent protein kinase, encoded by the Drosophila foraging gene, regulates neurotransmission through changes in synaptic structure and function. J Neurogenet. 35(3):213–220. doi:10.1080/01677063.2021.1905639.

Górska-Andrzejak J, Chwastek EM, Walkowicz L, Witek K. 2018. On Variations in the Level of PER in Glial Clocks of Drosophila Optic Lobe and Its Negative Regulation by PDF Signaling. Front Physiol. 9:230. doi:10.3389/fphys.2018.00230.

Hidalgo S, Anguiano M, Tabuloc CA, Chiu JC. 2023. Seasonal cues act through the circadian clock and pigment-dispersing factor to control EYES ABSENT and downstream physiological changes. Curr Biol. 33(4):675-687.e5. doi:10.1016/j.cub.2023.01.006.

Kayser MS, Yue Z, Sehgal A. 2014. A Critical Period of Sleep for Development of Courtship Circuitry and Behavior in Drosophila. Science. 344(6181):269–274. doi:10.1126/science.1250553.

Lee SG, Sun D, Miao H, Wu Z, Kang C, Saad B, Nguyen K-NH, Guerra-Phalen A, Bui D, Abbas A-H, et al. 2023. Taste and pheromonal inputs govern the regulation of time investment for mating by sexual experience in male Drosophila melanogaster. PLOS Genet. 19(5):e1010753. doi:10.1371/journal.pgen.1010753.

Reaume CJ, Sokolowski MB, Mery F. 2011. A natural genetic polymorphism affects retroactive interference in Drosophila melanogaster. Proc R Soc B: Biol Sci. 278(1702):91–98. doi:10.1098/rspb.2010.1337.

Renger JJ, Yao W-D, Sokolowski MB, Wu C-F. 1999. Neuronal Polymorphism among Natural Alleles of a cGMP-Dependent Kinase Gene, foraging , in Drosophila. J Neurosci. 19(19):RC28–RC28. doi:10.1523/jneurosci.19-19-j0002.1999.
