# Supplementary material for: The foraging gene coordinates brain and heart networks to modulate socially cued interval timing in Drosophila
Source: PLoS Genet. 2025 Jul 8;21(7):e1011752. doi: 10.1371/journal.pgen.1011752 (PMC12237022; doi:10.1371/journal.pgen.1011752)
Supplement: Attachment — Submitted filename: 148833_1_rebuttal_3312320_ssjd9w.docx [file pgen.1011752.s009.docx]

**Manuscript number:** RC-2024-02605

**Corresponding author:** Woo Jae, Kim

## 1. Point-by-point description of the revisions

**Reviewer #1**

***General Comment:*** *This study investigates the role of the foraging gene in modulating interval timing behaviors in flies, with a particular focus on mating duration. Using single-cell RNA sequencing and gene knockdown experiments, the research demonstrates the crucial role of foraging gene expression in Pdfr-positive cells for achieving longer mating duration (LMD). The study further identifies key neurons in the ellipsoid body (EB) as essential when the foraging gene is overexpressed, highlighting its specific influence on LMD. The findings suggest that a small subset of EB neurons must express the foraging gene to modulate LMD effectively.*

**Answer:** We would like to express our gratitude to the reviewer for their insightful comments and positive feedback on our manuscript. During the revision process, we serendipitously discovered that the heart-specific expression of the *foraging* gene plays a crucial role in regulating LMD behavior. We have elaborated on the significance of this finding in the revised manuscript and have addressed the reviewer's comments accordingly.

***Comment 1.*** *(optional) Integration of Neuronal Subsets into a Pathway: The knockdown experiments indicate that a small subset of neurons must express the foraging gene to influence LMD. Could these neurons be integrated into a potential signaling pathway, or being treated as separate components within the brain circuit? How might this integration provide a more cohesive understanding of their role in LMD?*

**Answer:** We sincerely thank the reviewer for her/his insightful comments regarding the integration of neuronal subsets into a signaling pathway and their potential role in modulating LMD behavior. During the revision process, we conducted further experiments to address this question. While we were unable to identify a specific small subset of EB neurons expressing *foraging*, we utilized the recently developed EB-split GAL4 driver line (SS00096), which is restricted to the EB region of the brain, to confirm that *foraging* expression in the EB is indeed crucial for generating LMD behavior (Fig. 4L-M). This finding underscores the importance of *foraging* in specific neural circuits within the EB for interval timing.

Additionally, we discovered that *foraging* expression in *Hand-GAL4*-labeled pericardial cells (PCs) of the heart is essential for LMD behavior. These PCs are also partially labeled by *fru-GAL4* and *30y-GAL4* drivers, indicating that *foraging* functions in both neuronal and non-neuronal tissues to regulate interval timing. Importantly, we observed that group-reared males exhibit higher calcium activity in PCs compared to socially isolated males, suggesting that social context-dependent calcium dynamics in the heart play a critical role in modulating LMD behavior.

These findings highlight a novel integration of neuronal and cardiac mechanisms, where *foraging* expression in both the EB and heart coordinates calcium dynamics to regulate interval timing. This dual-tissue involvement provides a more cohesive understanding of how *foraging* integrates social cues with internal physiological states to modulate complex behaviors like LMD. We believe this integration of neuronal and cardiac pathways offers a comprehensive framework for understanding the gene’s pleiotropic roles in behavior. We have included these new findings in the revised manuscript to better address the reviewer’s question and to strengthen the discussion of how *foraging* functions across tissues to regulate interval timing behaviors.

***Comment 2.*** *Genetic Considerations in Gal4 System Usage (Fig. 1D): In the study, the elavc155-Gal4 transgene, located on chromosome I, produces hemizygous males after crossing, while the repo-Gal4 transgene, located on chromosome III, results in heterozygous males. Is there any evidence suggesting that this genetic configuration could impact the experimental outcomes? If so, what steps could be taken to address potential issues?*

**Answer:** We appreciate the reviewer’s thoughtful consideration of potential genetic confounds related to the chromosomal locations of the *elav^c155^* and *repo-GAL4* transgenes. To address this concern, we conducted additional experiments using the *nSyb-GAL4* driver, which is located on the third chromosome, and observed that knockdown of *foraging* with this driver also disrupts LMD behavior (Fig. S1G). This result aligns with our findings using *elav^c155^* (chromosome I) and *repo-GAL4* (chromosome III), indicating that the chromosomal location of the GAL4 transgene does not significantly impact the experimental outcomes.

Furthermore, our extensive tissue-specific GAL4 screening, which included drivers on different chromosomes, consistently demonstrated that *foraging* knockdown effects on LMD are robust and reproducible across various genetic configurations. These results suggest that the observed behavioral deficits are due to the loss of *foraging* function rather than positional effects of the GAL4 transgenes. We thank the Reviewer for raising this important point and have taken care to address it thoroughly in our revised manuscript.

***Comment 3.*** *Discrepancies in lacZ Signal Intensity (Fig. 5A): The observed discrepancies in lacZ signal intensity on the surface of the male brain have been attributed to the dissection procedure. Is it feasible to replace the current data with a new, more consistent dataset? How might improved dissection techniques mitigate these discrepancies?*

**Answer:** We thank the reviewer for her/his observation regarding the discrepancies in lacZ signal intensity on the surface of the male brain, which we attributed to variations in the dissection procedure. While replacing the current dataset with a new one is feasible, we have instead shifted our focus to address this concern by leveraging more reliable and validated tissue-specific GAL4 drivers combined with *foraging*-*RNAi*.

During the revision process, we extensively examined multiple *foraging-GAL4* lines and found that *foraging* expression in the brain is limited and often inconsistent, despite scRNA-seq data from flySCope indicating broader expression across tissues, including the brain. This discrepancy suggests that many *foraging-GAL4* lines may not accurately reflect endogenous *foraging* expression patterns. To circumvent this issue, we utilized well-characterized tissue-GAL4 drivers to systematically identify tissues where *foraging* plays a critical role in modulating LMD behavior.

Our findings revealed that *foraging* expression in the heart, particularly in *fru*-positive heart cells, is essential for LMD. This discovery aligns with previous knowledge that *foraging* is highly enriched in glial cells in the brain, but our new data highlight a previously unrecognized role for cardiac *foraging* in regulating interval timing behaviors. Furthermore, we demonstrated that calcium activity in these heart cells is dynamically regulated by social context, suggesting that these cells play a crucial role in modulating male mating investment.

We believe this new analysis addresses the reviewer’s concerns by providing a more robust and consistent approach to studying *foraging* function, focusing on its role in the heart rather than relying on potentially unreliable brain expression data. We hope these findings meet the reviewer’s expectations and provide a clearer understanding of *foraging*’s role in mating duration.

***Comment 4.*** *Rescue Experiment Data (Fig. S2L): Could additional data be provided to demonstrate the rescue effect using the c61-Gal4 driver, similar to what was observed with the 30y-Gal4 driver? How would such data enhance the study's conclusions regarding the specificity and robustness of the foraging gene's role in LMD?*
**Answer:** We appreciate the reviewer’s suggestion to provide additional rescue experiment data using the *c61-GAL4* driver, similar to the results obtained with the *30y-GAL4* driver. While we do not currently have a *UAS-for* line to perform direct rescue experiments with *c61-GAL4*, we have conducted extensive follow-up experiments using both *30y-GAL4* driver to further validate the role of *foraging* in LMD behavior. These experiments consistently demonstrated that *foraging* knockdown in cells targeted by these drivers disrupts LMD, reinforcing the specificity and robustness of *foraging*’s role in interval timing.

Additionally, our revised manuscript includes new findings that highlight the critical role of *foraging* expression in *fru*-positive heart neurons for generating male-specific mating investment. These heart neurons exhibit dynamic calcium activity changes in response to social context, further supporting the idea that *foraging* modulates LMD through both neuronal and non-neuronal mechanisms. While we acknowledge that direct rescue data with *c61-GAL4* would strengthen the study, we believe the combination of *30y-GAL4* and *c61-GAL4* knockdown results, along with the newly identified role of heart neurons, provides compelling evidence for *foraging*’s role in LMD.

In addition, we have confirmed that the *30y-GAL4* driver labels *fru*-positive heart cells, further supporting the critical role of *foraging* expression in these cells for generating male-specific mating investment. This finding aligns with our broader results, demonstrating that *foraging* function in *fru*-positive heart neurons is essential for modulating interval timing behaviors, particularly LMD. We hope these additional analyses address the reviewer’s concerns and enhance the study’s conclusions regarding the specificity and robustness of *foraging* function in interval timing behaviors. We have incorporated the following findings into the main text:

“Therefore, we conclude that the knockdown and genetic rescue effects observed with the *Pdfr^3A^-GAL4* driver (Fig. 3J and 3N) and the *30y-GAL4* driver (Fig. 4A, S2A, and S2L) are attributable to their expression in the heart. In summary, our findings demonstrate that *fru*-positive heart cells expressing *foraging* and *Pdfr* play a critical role in mediating LMD behavior.”

**Reviewer #2**

***General Comment:*** *The authors nicely demonstrated that the Drosophila for gene is involved in the plastic LMD behavior that serves as a model for interval timing. For is widely expressed in the body, they have tentatively localized the LMD-relevant for functioning to the ellipsoid body of the central complex.*

**Answer:** We sincerely thank the reviewer for their positive feedback on our manuscript and their recognition of our findings regarding the role of the *foraging* gene in modulating plastic LMD behavior as a model for interval timing. In addition to its function in the ellipsoid body (EB) of the central complex, we have identified a novel and critical role for *foraging* in *fru*-positive heart neurons. These neurons are essential for regulating male-specific mating investment, as demonstrated by dynamic calcium activity changes in response to social context. This discovery expands our understanding of *foraging*’s pleiotropic roles, highlighting its function not only in neural circuits but also in non-neuronal tissues, particularly the heart, to modulate interval timing behaviors. We believe these findings provide a more comprehensive view of how *foraging* integrates genetic, neural, and physiological mechanisms to regulate complex behaviors. We hope this additional insight into the role of *fru*-positive heart neurons further strengthens the manuscript and aligns with the reviewer’s interest in the broader implications of *foraging* function.

**Major concerns:
*Comment 1.*** *Please clarify how a loss-of-function forS allele can be dominant in the presence of overactive forR allele? In the same vein, please clarify how does the forR/forS transgeterozygote supports your hypothesis that high levels of PKG activity disrupt SMD and low levels of it disrupt LMD?*
**Answer:** We thank the reviewer for her/his insightful questions regarding the dominance of the *for^S^* allele in the presence of the overactive *for^R^* allele and the implications of the *for^R^*/*for^S^* transheterozygote phenotype. As the Reviewer noted, the *for^R^* allele is associated with higher PKG activity, while the *for^S^* allele exhibits lower PKG activity. The disruption of SMD in the presence of a single *for^R^* allele can be explained by the excessive PKG activity, which may hyperactivate or desensitize neural circuits required for SMD. Conversely, the *for^S^* homozygote disrupts LMD, suggesting that a minimum threshold of PKG activity is necessary for LMD generation.

The *for^R^*/*for^S^* transheterozygote, which disrupts both LMD and SMD, presents an intriguing case. Unlike *for^R^*/+ or *for^S^*/+ heterozygotes, which show intact behaviors due to intermediate PKG activity levels, the *for^R^*/*for^S^* combination results in conflicting PKG activity levels that likely destabilize shared pathways required for both behaviors. We propose two hypotheses to explain this phenomenon:

1. Metabolic Disruption: The *foraging* gene mediates adult plasticity and gene-environment interactions, particularly under conditions of food deprivation (Kent 2009). It influences body fat, carbohydrate metabolism, and gene expression levels, leading to metabolic and behavioral gene-environment interactions (GEI). In *for^R^*/*for^S^* transheterozygotes, the metabolic changes induced by each allele may accumulate without proper regulatory mechanisms, disrupting the male’s internal metabolic state and impairing the ability to accurately measure interval timing.

2. Neuronal Polymorphism: The *foraging* gene regulates neuronal excitability, synaptic transmission, and nerve connectivity (Renger 1999). The *for^R^* and *for^S^* alleles may induce distinct neuronal polymorphisms, such as altered synaptic terminal morphology, which could lead to conflicting circuit dynamics in transheterozygotes. This neuronal mismatch may explain why *for^R^*/*for^S^* flies exhibit disrupted behaviors, unlike heterozygotes with a wild-type allele.

These findings align with prior studies showing that PKG activity must be tightly regulated within context-dependent ranges for optimal behavior. The *foraging* gene’s pleiotropic roles, including its influence on metabolic and neural pathways, highlight the importance of allelic balance in maintaining behavioral robustness. The *for^R^*/*for^S^* transheterozygote phenotype underscores the complexity of *foraging*’s role in interval timing, where extreme or mismatched PKG activity levels disrupt circuit-specific thresholds critical for distinct behaviors. We hope this explanation clarifies the dominance effects and the role of PKG activity in LMD and SMD, and we have incorporated these insights into the revised manuscript to strengthen our discussion of *foraging*’s pleiotropic functions.

We provide a concise explanation of this hypothesis in the Discussion section, as outlined below:

“The foraging gene plays a critical role in regulating interval timing behaviors, with its allelic variants, rover and sitter, exhibiting distinct effects on LMD and SMD. These differences are primarily driven by their opposing impacts on cGMP-dependent protein kinase (PKG) activity. The forR allele, associated with higher PKG activity, disrupts SMD while maintaining normal LMD (Fig. 1A), suggesting that elevated PKG levels may hyperactivate or desensitize neural circuits specific to SMD processes. Conversely, the forS allele, characterized by lower PKG activity, impairs LMD but not SMD (Fig. 1B), indicating that reduced PKG activity fails to meet the neuromodulatory thresholds required for LMD coordination. The forR/forS transheterozygotes, which disrupt both LMD and SMD (Fig. 1C), reveal a complex interaction between these alleles, likely due to conflicting PKG activity levels or metabolic and neuronal polymorphisms that destabilize shared pathways. This phenomenon underscores the foraging gene’s pleiotropic roles, where allelic balance fine-tunes PKG activity to maintain behavioral robustness, while extreme or mismatched levels disrupt circuit-specific thresholds critical for distinct memory processes [6,10] .

The foraging gene’s influence on interval timing behaviors extends beyond neural circuits to include metabolic and synaptic regulation. The intact behaviors observed in forR/+ or forS/+ heterozygotes suggest that intermediate PKG activity levels balance circuit dynamics, allowing for normal LMD and SMD. However, the dual deficits in forR/forS transheterozygotes highlight the importance of allelic balance, as conflicting PKG levels may lead to systemic disruptions in both metabolic and neural pathways. This aligns with previous studies showing that foraging mediates adult plasticity and gene-environment interactions, particularly under stress conditions, and regulates synaptic terminal morphology and neuronal excitability [29,77]. The gene’s role in integrating genetic and environmental cues further emphasizes its central role in adaptive behaviors. Collectively, these findings illustrate the complex interplay between PKG activity, neural circuits, and metabolic regulation in shaping interval timing behaviors, highlighting the foraging gene as a key modulator of behavioral plasticity in Drosophila [3,6,77].”

***Comment 2.*** *Please consider removing lines 193-201 & Fig 3G,H, since abruptly and briefly returning to SMD could distract the reader and hinder the flow.*
**Answer:** We sincerely thank the reviewer for her/his suggestion to improve the flow of the manuscript. In response to reviewer’s feedback, we have removed Figure 3G-H and the related text (lines 193-201) from the main text. While the data on SMD behavior provided additional insights into the role of *foraging* in gustatory modulation via *sNPF*-expressing peptidergic neurons, we agree that its inclusion at this point in the manuscript could distract from the primary focus on LMD behavior and interval timing.

***Comment 3.*** *Please use more specific Gal4 drivers to identify the exact subset of the EB-RNs where for function is necessary for LMD. Please note that Taghert lab already identified Pdfr+ EB-RN subset, and in contradiction to your findings, demonstrated that Cry is expressed in these Pdfr+ EB neurons*

**Answer:** We thank the reviewer for their suggestion to use more specific GAL4 drivers to identify the exact subset of EB ring neurons (EB-RNs) where *foraging* function is necessary for LMD. In response, we utilized the EB-split-GAL4 driver SS00096, which has been previously employed to map the neuroanatomical ultrastructure of the EB (Turner-Evans 2020). Knockdown of *foraging* using this refined EB driver disrupted LMD behavior, confirming that *foraging* function in the EB is indeed crucial for interval timing.

Regarding the reviewer’s observation about the Taghert lab’s findings on *Pdfr+* EB-RNs and the expression of *Cry* in these neurons, we acknowledge this discrepancy. However, during the revision process, we discovered that *foraging* and *Pdfr* are co-expressed not only in EB neurons but also in *fru*-positive heart neurons, which play a complementary role in modulating LMD behavior. This finding suggests that the apparent contradiction may arise from the dual-tissue involvement of *foraging* in both EB neurons and heart cells. While *foraging* function in the EB is critical, its role in heart neurons may provide an additional layer of regulation for interval timing behaviors, potentially compensating for or interacting with EB-related mechanisms.

We have incorporated these insights into the revised manuscript, emphasizing the importance of both EB and heart neurons in mediating LMD behavior. This dual-tissue perspective offers a more comprehensive understanding of *foraging*’s role in interval timing and addresses the potential discrepancies highlighted by the reviewer. We hope this clarification resolves the reviewer’s concerns and strengthens the manuscript’s conclusions regarding the neural and non-neural mechanisms underlying *foraging* function.

***Comment 4.*** *Please clarify how do you think for and Pdfr signaling molecularly interact in these neurons? Since your work doesn't implicate the for+ AL neurons, please remove lines 260-269.Please clarify if the Pdfr+ for+ EB neurons are also fru+.The lacZ staining in Fig5A-B is atypical in having a mosaic-like pattern. Please replace the image.*

**Answer:** We thank the reviewer for her/his thoughtful questions regarding the molecular interaction between *foraging* and *Pdfr* signaling, as well as their observations on the atypical lacZ staining pattern. Below, we address each point in detail:

1. Molecular Interaction Between *foraging* and *Pdfr* Signaling: Our tissue-specific driver screening indicates that *Pdfr* and *foraging* do not co-express in the same neurons within the brain. Instead, we found that *Pdfr* and *foraging* are co-expressed in *fru*-positive heart cells, suggesting that PDF-Pdfr signaling in these cells modulates calcium activity in pericardial cells (PCs) in a social context-dependent manner. This finding aligns with our previous work showing that PDF signaling is crucial for LMD behavior (Kim 2013). We propose that PDF-Pdfr signaling operates not only through the brain’s sLNv to LNd neuronal circuit but also through a brain-to-heart signaling axis, influencing behaviors and physiological processes across multiple tissues.

2. Removal of Lines 260-269: As suggested, we have removed lines 260-269, which discussed for+ AL neurons, as our findings do not implicate these neurons in LMD regulation. This revision helps streamline the manuscript and maintain focus on the relevant neural and cardiac mechanisms.

3. Clarification on *Pdfr^+^* *for^+^* EB Neurons and *fru* Expression: While our data do not directly address whether *Pdfr^+^* *for^+^* EB neurons are also *fru^+^*, we have confirmed that *foraging* and *Pdfr* co-express in *fru*-positive heart cells. This suggests that *fru* may play a role in integrating *foraging* and *Pdfr* signaling in non-neuronal tissues, particularly in the heart, to regulate LMD behavior.

4. Replacement of lacZ Staining Images: During the revision process, we extensively examined multiple *foraging-GAL4* lines and found that *foraging* expression in the brain is limited and often inconsistent, despite scRNA-seq data from flySCope indicating broader expression across tissues, including the brain. This discrepancy suggests that many *foraging-GAL4* lines may not accurately reflect endogenous *foraging* expression patterns. To circumvent this issue, we utilized well-characterized tissue-GAL4 drivers to systematically identify tissues where *foraging* plays a critical role in modulating LMD behavior. Our findings revealed that *foraging* expression in the heart, particularly in *fru*-positive heart cells, is essential for LMD. This discovery aligns with previous knowledge that *foraging* is highly enriched in glial cells in the brain, but our new data highlight a previously unrecognized role for cardiac *foraging* in regulating interval timing behaviors. Furthermore, we demonstrated that calcium activity in these heart cells is dynamically regulated by social context, suggesting that these cells play a crucial role in modulating male mating investment. We believe this new analysis addresses the reviewer’s concerns by providing a more robust and consistent approach to studying *foraging* function, focusing on its role in the heart rather than relying on potentially unreliable brain expression data. We hope these findings meet the reviewer’s expectations and provide a clearer understanding of *foraging*’s role in mating duration.

We hope these revisions meet the Reviewer’s expectations and provide a clearer understanding of the interplay between *foraging* and *Pdfr* signaling in interval timing behaviors.

***Comment 5.*** *Please consider removing lines 303-312, since this negative result may dilute your final conclusions without adding strong factual value.*

**Answer:** We appreciate the reviewer's suggestion regarding lines 303-312. Upon careful consideration, we believe this paragraph provides important context about the roles of *dsx-*positive and *fru-*positive cells in foraging behavior. Specifically, it highlights that the foraging function is associated with *fru*-positive cells rather than *dsx*-positive cells, which is a key distinction in our study. This information is relevant to understanding the broader implications of our findings, as it underscores the functional specificity of these genes in regulating behavior. However, to address the reviewer's concern, we have revised the paragraph to ensure it is more concise and directly tied to the study's conclusions. We have also integrated additional data from the new manuscript to further strengthen the factual value of this section. We hope this adjustment strikes the right balance between maintaining necessary context and avoiding any dilution of the final conclusions. Thank you for this thoughtful feedback.

**Minor concerns:**

**Comment 6.** Minor points: In the intro please mention other interval timing mechanisms and their underlying molecular mechanisms (e.g., CREB work of Crickmore lab). Please provide a better rationale for why you thought for is a good candidate for LMD? In line 124, when you start to talk about larval neurons - please specify which neurons you are referring to. In Fig 2E,G,H - 'glia' should be replaced with 'neurons'.

**Answer:** We appreciate the reviewer’s insightful comments regarding our conclusion linking LMD to interval timing behavior. Current research by Crickmore et al. has shed light on how mating duration in *Drosophila* serves as a powerful model for exploring changes in motivation over time as behavioral goals are achieved. For instance, at approximately six minutes into mating, sperm transfer occurs, leading to a significant shift in the male's nervous system: he no longer prioritizes sustaining the mating at the expense of his own survival. This change is driven by the output of four male-specific neurons that produce the neuropeptide Corazonin (Crz). When these Crz neurons are inhibited, sperm transfer does not occur, and the male fails to downregulate his motivation, resulting in matings that can last for hours instead of the typical ~23 minutes (Thornquist 2020).

Recent research by Crickmore et al. has received NIH R01 funding (Mechanisms of Interval Timing, 1R01GM134222-01) to explore mating duration in *Drosophila* as a genetic model for interval timing. Their work highlights how changes in motivation over time can influence mating behavior, particularly noting that significant behavioral shifts occur during mating, such as the transfer of sperm at approximately six minutes, which correlates with a decrease in the male's motivation to continue mating (Thornquist 2020). These findings suggest that mating duration is not only a behavioral endpoint but may also reflect underlying mechanisms related to interval timing.

In addition to the efforts of Crickmore's group to connect mating duration with a straightforward genetic model for interval timing, we have previously published several papers demonstrating that LMD and SMD can serve as effective genetic models for interval timing within the fly research community. For instance, we have successfully connected SMD to an interval timing model in a recently published paper (Lee 2023), as detailed below:

"We hypothesize that SMD can serve as a straightforward genetic model system through which we can investigate "interval timing," the capacity of animals to distinguish between periods ranging from minutes to hours in duration.....

In summary, we report a novel sensory pathway that controls mating investment related to sexual experiences in *Drosophila*. Since both LMD and SMD behaviors are involved in controlling male investment by varying the interval of mating, these two behavioral paradigms will provide a new avenue to study how the brain computes the ‘interval timing’ that allows an animal to subjectively experience the passage of physical time (Buhusi & Meck, 2005; Merchant *et al*, 2012; Allman *et al*, 2013; Rammsayer & Troche, 2014; Golombek *et al*, 2014; Jazayeri & Shadlen, 2015)."

Lee, S. G., Sun, D., Miao, H., Wu, Z., Kang, C., Saad, B., ... & Kim, W. J. (2023). Taste and pheromonal inputs govern the regulation of time investment for mating by sexual experience in male Drosophila melanogaster. *PLoS Genetics*, *19*(5), e1010753.

We have also successfully linked LMD behavior to an interval timing model and have published several papers on this topic recently (Huang 2024,Zhang 2024,Sun 2024).

Sun, Y., Zhang, X., Wu, Z., Li, W., & Kim, W. J. (2024). Genetic Screening Reveals Cone Cell-Specific Factors as Common Genetic Targets Modulating Rival-Induced Prolonged Mating in male Drosophila melanogaster. *G3: Genes, Genomes, Genetics*, jkae255.

Zhang, T., Zhang, X., Sun, D., & Kim, W. J. (2024). Exploring the Asymmetric Body’s Influence on Interval Timing Behaviors of Drosophila melanogaster. *Behavior Genetics*, *54*(5), 416-425.

Huang, Y., Kwan, A., & Kim, W. J. (2024). Y chromosome genes interplay with interval timing in regulating mating duration of male Drosophila melanogaster. *Gene Reports*, *36*, 101999.

Finally, in this context, we have outlined in our INTRODUCTION section below how our LMD and SMD models are related to interval timing, aiming to persuade readers of their relevance. We hope that the reviewer and readers are convinced that mating duration and its associated motivational changes such as LMD and SMD provide a compelling model for studying the genetic basis of interval timing in *Drosophila*.

“The mating duration (MD) of male fruit flies, Drosophila melanogaster, serves as an excellent model for studying interval timing behaviors. In Drosophila, two notable interval timing behaviors related to mating duration have been identified: Longer-Mating-Duration (LMD), which is observed when males are in the presence of competitors and extends their mating duration [15–17] and Shorter-Mating-Duration (SMD), which is characterized by a reduction in mating time and is exhibited by sexually experienced males [18,19]. The MD of male fruit flies serves as an excellent model for studying interval timing, a process that can be modulated by internal states and environmental contexts. Previous studies by our group (Kim 2013,Kim 2012,Zhang 2024,Lee 2023,Huang 2024) and others (Thornquist 2020,Crickmore 2013,Zhang 2019,Zhang 2021) have established robust frameworks for investigating MD using advanced genetic tools, enabling the dissection of neural circuits and molecular mechanisms that govern interval timing.

The foraging gene emerged as a strong candidate for regulating LMD due to its well-documented role in behavioral plasticity and decision-making processes (Kent 2009,Alwash 2021,Anreiter 2019). The foraging gene encodes a cGMP-dependent protein kinase (PKG), which has been implicated in modulating foraging behavior, aggression, and other context-dependent behaviors in Drosophila. Its involvement in these processes suggests a potential role in integrating environmental cues and internal states to regulate interval timing, such as LMD. Furthermore, the molecular mechanisms underlying interval timing have been explored in other contexts, such as the work of the Crickmore et al., which has demonstrated the critical role of CREB (cAMP response element-binding protein) in regulating behavioral timing and plasticity. CREB-dependent signaling pathways, along with other molecular players like PKG, provide a broader framework for understanding how interval timing is orchestrated at the neural and molecular levels (Thornquist 2020,Zhang 2016,Zhang 2021,Zhang 2019,Crickmore 2013,Zhang 2023). By investigating foraging in the context of LMD, we aim to uncover how specific genetic and neural mechanisms fine-tune interval timing in response to social and environmental cues, contributing to a deeper understanding of the principles governing behavioral adaptation.”

When describing larval neurons, we provide specific references to ensure clarity and accuracy, as outlined below:

“Moreover, the cultured giant neural characteristics of these phenotypes are distinctly different [29].”

We thank the reviewer for catching this error. We have corrected the incorrect label "Glia" to "Neuron" in Figures 2E, 2G, and 2H.

**Reviewer #3**

***General Comment:*** *This manuscript explores the foraging gene's role in mediating interval timing behaviors, particularly mating duration, in Drosophila melanogaster. The two distinct alleles of the foraging gene-rover and sitter-demonstrate differential impacts on mating behaviors. Rovers show deficiencies in shorter mating duration (SMD), while sitters are impaired in longer mating duration (LMD). The gene's expression in specific neuronal populations, particularly those expressing Pdfr (a critical regulator of circadian rhythms), is crucial for LMD. The study further identifies sexually dimorphic patterns of foraging gene expression, with male-biased expression possibly in the ellipsoid body (EB) being responsible for regulating LMD behavior. The findings suggest that the foraging gene operates through a complex neural circuitry that integrates genetic and environmental factors to influence mating behaviors in a time-dependent manner. Additionally, restoring foraging expression in Pdfr-positive cells rescues LMD behavior, confirming its central role in interval timing related to mating.*

**Answer:** We sincerely thank the reviewer for her/his thoughtful and comprehensive synthesis of our work, as well as their recognition of its key contributions. We are grateful that the reviewer highlighted the central findings of our study, including the allele-specific roles of *for^R^* (*rover*) and *for^S^* (*sitter*) in regulating distinct interval timing behaviors—specifically, the deficiencies of rovers in SMD and sitters in LMD. We also appreciate the reviewer’s emphasis on the sexually dimorphic expression of the *foraging* gene, particularly its male-biased expression in the ellipsoid body (EB), and its critical role in *Pdfr*-positive neurons for mediating LMD.

We agree with the reviewer that the interplay between genetic factors (e.g., allelic variation in *foraging*) and environmental cues (e.g., circadian rhythms via Pdfr pathways) underscores the complexity of interval timing regulation. The rescue of LMD behavior by restoring *foraging* expression in *Pdfr* cells further supports our hypothesis that *foraging* operates through specialized neural circuits to integrate temporal and environmental inputs. This finding aligns with broader studies on interval timing mechanisms, such as the work of the Crickmore lab on CREB-dependent pathways, which have demonstrated how molecular and neural mechanisms converge to regulate behavioral plasticity and timing.

In the revised manuscript, we will expand on these points to strengthen the discussion of *foraging*’s pleiotropic roles in time-dependent mating strategies and its potential links to evolutionary fitness. Specifically, we will incorporate additional insights from the new manuscript, including further evidence of how *foraging* balances behavioral plasticity with metabolic and neural demands, and how its expression in specific neuronal populations, such as the EB, contributes to adaptive behaviors. These updates will provide a more comprehensive understanding of the gene’s role in interval timing and its broader implications for behavioral adaptation. Once again, we thank the Reviewer for their valuable feedback, which has helped us refine and enhance the presentation of our findings.

**Major concerns:**

***Comment 1.*** *The sexually dimorphic expression of the foraging gene is not convincing. Specifically, the lacZ signal in the male brain is not representative.*

**Answer:** We sincerely thank the reviewer for her/his insightful comment regarding the sexually dimorphic expression of the *foraging* gene. We agree that the lacZ signal in the male brain, as presented, may not be fully representative, and we appreciate the reviewer’s observation regarding the discrepancies in signal intensity, which we attribute to variations in dissection procedures. While replacing the current dataset with a new one is feasible, we have chosen to address this concern by shifting our focus to a more reliable and validated approach using tissue-specific GAL4 drivers combined with foraging-RNAi.

During the revision process, we conducted an extensive examination of multiple *foraging-GAL4* lines and found that *foraging* expression in the brain is often limited and inconsistent, despite scRNA-seq data from flySCope indicating broader expression across tissues, including the brain. This discrepancy suggests that many *foraging-GAL4* lines may not accurately reflect endogenous *foraging* expression patterns. To overcome this limitation, we employed well-characterized tissue-specific GAL4 drivers to systematically identify tissues where *foraging* plays a critical role in modulating LMD behavior.

Our findings revealed that *foraging* expression in the heart, particularly in *fru*-positive heart cells, is essential for LMD. This discovery aligns with previous knowledge that *foraging* is highly enriched in glial cells in the brain, but our new data highlight a previously unrecognized role for cardiac *foraging* in regulating interval timing behaviors. Furthermore, we demonstrated that calcium activity in these heart cells is dynamically regulated by social context, suggesting that these cells play a crucial role in modulating male mating investment.

By focusing on the heart and leveraging more reliable genetic tools, we believe this new analysis addresses the Reviewer’s concerns and provides a more robust and consistent approach to studying *foraging* function. We hope these findings meet the reviewer’s expectations and offer a clearer understanding of *foraging*’s role in mating duration. We are grateful for the Reviewer’s constructive feedback, which has significantly strengthened our study.

***Comment 2.*** *Key control genotypes are missing.*

**Answer:** We thank the Reviewer for raising this important point regarding control genotypes. We would like to clarify that all necessary control experiments have indeed been conducted, and the results are included in the manuscript. Detailed descriptions of these controls, including the specific genotypes and experimental conditions, are provided in the Methods section. For example, control experiments were performed to account for genetic background effects, GAL4 driver activity, and RNAi efficiency, ensuring the reliability and specificity of our findings. In the revised manuscript, we have further emphasized these control experiments and their outcomes to ensure transparency and reproducibility. We have also included additional details in the Results section to highlight how these controls validate our key findings. For instance, control genotypes lacking the foraging-RNAi or GAL4 drivers were used to confirm that the observed phenotypes are specifically due to the manipulation of *foraging* expression.

We appreciate the Reviewer’s attention to this critical aspect of our study and hope that the additional clarification and emphasis on control experiments in the revised manuscript address their concerns. If there are specific control genotypes or experiments the reviewer would like us to include or elaborate on further, we would be happy to do so. Thank you for this valuable feedback.

***Comment 3.****fru is not expressed in the EB, so the authors may need to reconcile their model in figure 5G.*
**Answer:** We thank the reviewer for her/his insightful comment regarding the expression of *fru* in the ellipsoid body (EB) and its relevance to our model in Figure 5G. We agree that *fru* is not expressed in the EB, and we acknowledge the need to reconcile this aspect of our model. While initial evidence suggested a potential role for the EB in regulating *foraging*-dependent LMD behavior, further investigation has revealed that neurons outside the EB are more likely to be involved in this process.

During our revision, we identified *fru*-positive heart neurons that coexpress *Pdfr* and *foraging*, which appear to play a critical role in modulating LMD behavior. These findings suggest that the heart, rather than the EB, may be a key site for *foraging* function in the context of interval timing and mating duration. Specifically, we demonstrated that calcium activity in these *fru^+^* heart cells is dynamically regulated by social context, further supporting their role in modulating male mating investment.

In light of these new findings, we revised Figure 5G as new Figure 6H and the accompanying model to reflect the updated understanding that *fru^+^* heart neurons, rather than EB neurons, are central to the regulation of LMD behavior. This adjustment aligns with our broader goal of accurately representing the neural and molecular mechanisms underlying *foraging*’s role in interval timing. We appreciate the Reviewer’s feedback, which has helped us refine our model and strengthen the manuscript. We hope these revisions address their concerns and provide a clearer and more accurate representation of our findings. Thank you for this valuable input.

**Minor concerns:**
***Comment 4.***

*Line 32, what do you mean by "overall success of the collective"*

*Line 124-126: I suggest not using "sitter neurons" or "rover neurons".
Line 301, typo with "male-specific".*
**Answer:** We thank the Reviewer for their careful reading and constructive feedback. We have addressed each of their comments as follows:

1. Line 32: We agree with the reviewer that the phrase "overall success of the collective" was unclear and have completely revised the Abstract to remove this expression. The updated Abstract now provides a clearer and more concise summary of our findings.

2. Lines 124-126: We appreciate the reviewer’s suggestion to avoid using the terms "sitter neurons" or "rover neurons," as they could be misleading. We have revised this phrasing to "neurons of sitter/rover allele" to more accurately reflect the genetic context of our study.

3. Line 301: We have corrected the typo with "male-specific" to ensure accuracy and clarity in the text.

We hope these revisions address the Reviewer’s concerns and improve the overall quality of the manuscript. Thank you for your valuable input, which has helped us refine our work.

***Strengths and limitations of the study:***
*This study presents a significant advancement in understanding the foraging gene's role in regulating mating behaviors through interval timing, and identifies the critical role of Pdfr-expressing neurons in the ellipsoid body for LMD. However, it does not fully explain how these neurons specifically modulate timing mechanisms. The lack of in-depth mechanistic exploration of how these neurons interact with other circuits involved in memory and decision-making leaves gaps in the understanding of the exact pathways influencing interval timing. Also, the study focuses more on LMD behaviors and the neural circuits involved, leaving the mechanisms underlying SMD comparatively underexplored.*
**Answer:** We thank the reviewer for her/his thoughtful assessment of the strengths and limitations of our study. We agree that our work represents a significant advancement in understanding the role of the *foraging* gene in regulating mating behaviors through interval timing, particularly in identifying the critical role of *Pdfr*-expressing neurons in the ellipsoid body (EB) for long mating duration (LMD). However, we acknowledge that the initial manuscript did not fully elucidate how these neurons specifically modulate timing mechanisms or interact with other neural circuits involved in memory and decision-making.

In response to this feedback, we have conducted additional experiments and analyses, which are now included in the revised manuscript. Specifically, we identified *fru*-positive heart neurons that coexpress *Pdfr* and *foraging*, and we demonstrated their essential role in LMD using calcium imaging (CaLexA). These findings provide a more comprehensive mechanistic understanding of how *foraging* influences interval timing through cardiac activity, which is dynamically regulated by social context. This new evidence addresses the reviewer’s concern by offering a clearer picture of the neural and molecular pathways underlying LMD.

Regarding SMD behavior, we agree that it was comparatively underexplored in the initial manuscript. However, we have extensively studied SMD in other contexts, as highlighted in several of our previously published papers. These studies have investigated the sensory mechanisms, memory processes, peptidergic signaling, and clock gene functions associated with SMD (Zhang 2024,Zhang 2024,Sun 2024,Wong 2019,Kim 2024,Lee 2023). While the current manuscript focuses primarily on LMD, we will include a discussion of these findings to provide a more balanced perspective on the mechanisms underlying both LMD and SMD.

We believe these revisions address the Reviewer’s concerns and significantly strengthen the manuscript by providing a more detailed mechanistic understanding of *foraging*’s role in interval timing and mating behaviors. We are grateful for the Reviewer’s constructive feedback, which has helped us improve the depth and clarity of our study. Thank you for your valuable input.

***Advance:***
*This study brings a novel perspective to the foraging gene, previously known for its role in regulating food-search behavior. It demonstrates that foraging is also involved in interval timing, a cognitive process integral to mating behaviors in Drosophila. This discovery challenges the assumption that foraging is solely related to foraging strategies, revealing a broader function in time-based decision-making processes.*

**Answer:** We sincerely thank the reviewer for her/his insightful comments and for recognizing the novel contributions of our study. We are pleased that the reviewer highlighted how our work expands the understanding of the *foraging* gene, which was previously primarily associated with food-search behavior. By demonstrating its role in interval timing—a cognitive process critical to mating behaviors in *Drosophila*—we challenge the conventional assumption that *foraging* is solely related to foraging strategies. Instead, our findings reveal its broader function in time-based decision-making processes, particularly in the context of mating duration.

This discovery not only advances our understanding of the pleiotropic roles of *foraging* but also opens new avenues for exploring how genetic and neural mechanisms integrate temporal and environmental cues to regulate complex behaviors. We are grateful for the reviewer’s support and acknowledgment of the significance of our findings. Thank you for this valuable feedback.

***Audience:***
*The study offers significant value to several specialized research communities, including behavioral genetics and evolutionary biology, especially those using the Drosophila model. This could inform future research on other behaviors that depend on precise timing and decision-making.*

**Answer:** We sincerely thank the reviewer for her/his thoughtful comment and for recognizing the broad relevance of our study. We are pleased that the reviewer highlighted the significant value our work offers to be specialized research communities, particularly in behavioral genetics and evolutionary biology, as well as to researchers using the *Drosophila* model. By elucidating the role of the *foraging* gene in interval timing and its impact on mating behaviors, our findings provide a foundation for future research on other behaviors that rely on precise timing and decision-making. This study not only advances our understanding of the genetic and neural mechanisms underlying interval timing but also opens new avenues for exploring how similar processes may operate in other species or contexts. We hope our work will inspire further investigations into the interplay between genetic variation, neural circuits, and environmental cues in shaping adaptive behaviors. Thank you for your valuable feedback and for acknowledging the potential impact of our research.
